# Feasibility of Repeated Assessment of Cognitive Function in Older Adults Using a Wireless, Mobile, Dry-EEG Headset and Tablet-Based Games

**DOI:** 10.3389/fpsyt.2021.574482

**Published:** 2021-06-25

**Authors:** Esther C. McWilliams, Florentine M. Barbey, John F. Dyer, Md Nurul Islam, Bernadette McGuinness, Brian Murphy, Hugh Nolan, Peter Passmore, Laura M. Rueda-Delgado, Alison R. Buick

**Affiliations:** ^1^Cumulus Neuroscience Ltd, Belfast, United Kingdom; ^2^Cumulus Neuroscience Ltd, Dublin, Ireland; ^3^Centre for Public Health, School of Medicine, Dentistry and Biomedical Sciences, Queen's University Belfast, Belfast, United Kingdom; ^4^School of Electronics, Electrical Engineering and Computer Science, Queen's University Belfast, Belfast, United Kingdom; ^5^Trinity Centre for Biomedical Engineering, Trinity College, The University of Dublin, Dublin, Ireland

**Keywords:** EEG, EEG biomarker, cognition, gamification, mobile EEG

## Abstract

Access to affordable, objective and scalable biomarkers of brain function is needed to transform the healthcare burden of neuropsychiatric and neurodegenerative disease. Electroencephalography (EEG) recordings, both resting and in combination with targeted cognitive tasks, have demonstrated utility in tracking disease state and therapy response in a range of conditions from schizophrenia to Alzheimer's disease. But conventional methods of recording this data involve burdensome clinic visits, and behavioural tasks that are not effective in frequent repeated use. This paper aims to evaluate the technical and human-factors feasibility of gathering large-scale EEG using novel technology in the home environment with healthy adult users. In a large field study, 89 healthy adults aged 40–79 years volunteered to use the system at home for 12 weeks, 5 times/week, for 30 min/session. A 16-channel, dry-sensor, portable wireless headset recorded EEG while users played gamified cognitive and passive tasks through a tablet application, including tests of decision making, executive function and memory. Data was uploaded to cloud servers and remotely monitored via web-based dashboards. Seventy-eight participants completed the study, and high levels of adherence were maintained throughout across all age groups, with mean compliance over the 12-week period of 82% (4.1 sessions per week). Reported ease of use was also high with mean System Usability Scale scores of 78.7. Behavioural response measures (reaction time and accuracy) and EEG components elicited by gamified stimuli (P300, ERN, Pe and changes in power spectral density) were extracted from the data collected in home, across a wide range of ages, including older adult participants. Findings replicated well-known patterns of age-related change and demonstrated the feasibility of using low-burden, large-scale, longitudinal EEG measurement in community-based cohorts. This technology enables clinically relevant data to be recorded outside the lab/clinic, from which metrics underlying cognitive ageing could be extracted, opening the door to potential new ways of developing digital cognitive biomarkers for disorders affecting the brain.

## Introduction

Recent advances in digital technologies provide a wealth of opportunity in the management of health conditions. In neurological disease the heterogeneity and complexity of conditions, along with continuing reliance on traditional subjective measurement tools, have presented a challenge for the development of data-driven biomarkers for diagnosis, monitoring and prediction of therapeutic response (1–7). The suite of tools described in this paper was designed to enable longitudinal, in-home data collection of brain electrophysiology and cognitive performance. The platform comprises (1) a dry sensor, wireless electroencephalography (EEG) headset that records brain activity, (2) gamified versions of cognitive tasks, and (3) cloud-based storage and automatic processing—with the aim of identifying potential digital biomarkers with utility in neuropsychiatric and neurodegenerative disorders.

EEG directly reflects neural synaptic function, with similar patterns from animal to human (8–10) and thus has substantial potential as a brain-based, translatable biomarker for diseases such as schizophrenia (11–18), depression (19–21) and Alzheimer's disease (AD) (22–29). However, traditional research EEG setups are effortful and time-consuming, requiring expensive equipment and the support of personnel with technical training. Single or infrequent lab-based EEG recording sessions may be affected by a range of factors including fluctuations in levels of participants' mental alertness, fatigue and task-induced mental workload (30, 31). Similarly, cognition as traditionally measured in therapeutic research and practise tends to take the form of clinician administered batteries of neuropsychological tests [e.g., (32, 33)] which, whilst low burden and relatively inexpensive, are subject to variability in scores on repeated testing occasions (34). Infrequent, “snapshot” assessments are subject to measurement error arising from multiple factors, such as practise effects (35–37), the “white-coat effect” related to anxiety about suspected cognitive impairment (38), and day-to-day fluctuations in context (39), in mood and in perceived stress (40–44).

The adoption of modern technology into medicine allows for more innovative forms of data collection (e.g., wearable devices), increasing objectivity and taking advantage of powerful analytical tools to probe complex diseases. Further, digital tools may allow for more frequent sampling and detection of subtle daily fluctuations, at minimal disruption to the patient since data may be collected both inside and outside of the clinic. Progress in modern electronics and dry sensor technology means that EEG is emerging from amongst standard brain imaging methods as a mobile technology, suitable for deployment to very large cohorts for convenient at-home use (45). Likewise, neuropsychological testing can now be completed outside of the clinic through the use of automated, web-based assessments (46–49).

Mobile EEG systems are advancing quickly. Several studies have shown that it is possible in principle to collect EEG recordings using consumer-grade hardware, and from the data extract potentially useful neuronal signals, including spectral band-power measures (45, 50, 51) and task-evoked event-related potentials (ERPs) (52–55). However, studies using such devices have typically required some specialist equipment (e.g., a computer running bespoke software to present stimuli and record EEG), and a specialist experimenter to set up and supervise the recording. In addition, most consumer-grade EEG platforms operate using low numbers of electrodes, leaving some research questions and certain types of analysis out of reach for researchers. To the authors' knowledge, there exists no prior example of large-scale, unsupervised in-home, repeated sampling ERP research using a dry-sensor, portable, user-friendly EEG platform.

Innovative solutions can be deployed to enable us to carry out unsupervised data collection without placing undue burden on the user, such as ‘dry' sensors (i.e., eschewing the conductive gel used in the laboratory in favour of an easier electrode setup) and automated user-facing stepwise tutorials and notifications (to compensate for reduced environmental control outside the laboratory). Similarly, for use at home over repeated sessions, EEG/ERP tasks as used in research may not be particularly exciting or motivational for the user, but applying gamification can make these tasks more engaging and rewarding for participants (56) and gamified cognitive tasks can facilitate global data gathering on an unparalleled scale (57).

The study presented here was a first, proof-of-concept, field study to test the human-factors and technical feasibility of an early version of the Cumulus Neuroscience platform in a cohort of healthy adults spanning an age range up to 79 years old. In this paper we investigate the potential of this platform to capture in-home, frequent repeated measurement of EEG and behavioural metrics of cognitive ageing, metrics that also have broader appeal as potential cognitive biomarkers for the diagnosis and treatment of disorders affecting the brain. Use of the platform on a regular basis over 3 months assesses the acceptability of long-term use for future use cases where longitudinal progression tracking is required, avoids dependence on a single “snapshot” measurement, and allows for improved signal quality through aggregation of EEG data collected in the home (an unsupervised environment).

Analyses are presented that quantify reported ease-of-use, and resultant levels of weekly adherence over a period of 3 months of unsupervised at-home use. The gamified cognitive tasks are evaluated for face-validity, by comparing key known behavioural effects with data gathered in the home, and examining effects of age that have been reported in the literature. Similarly the EEG data is examined at grand-average level to confirm that it replicates the main features (waveform morphology and timing, frequency content, scalp topography) of the neural signatures that the gamified tasks are designed to elicit.

## Method

### Participants

89 healthy adult volunteers (67 female), aged between 40 and 79 years (mean 58.78, s.d. 8.86) with a Montreal Cognitive Assessment (MoCA) score ≥24 gave informed consent to take part in the procedures approved by Queen's University Belfast Ethics Committee. Recruitment channels included “Join Dementia Research,” local community groups and use of print media and social media.

### The Platform

**Figure 1:**
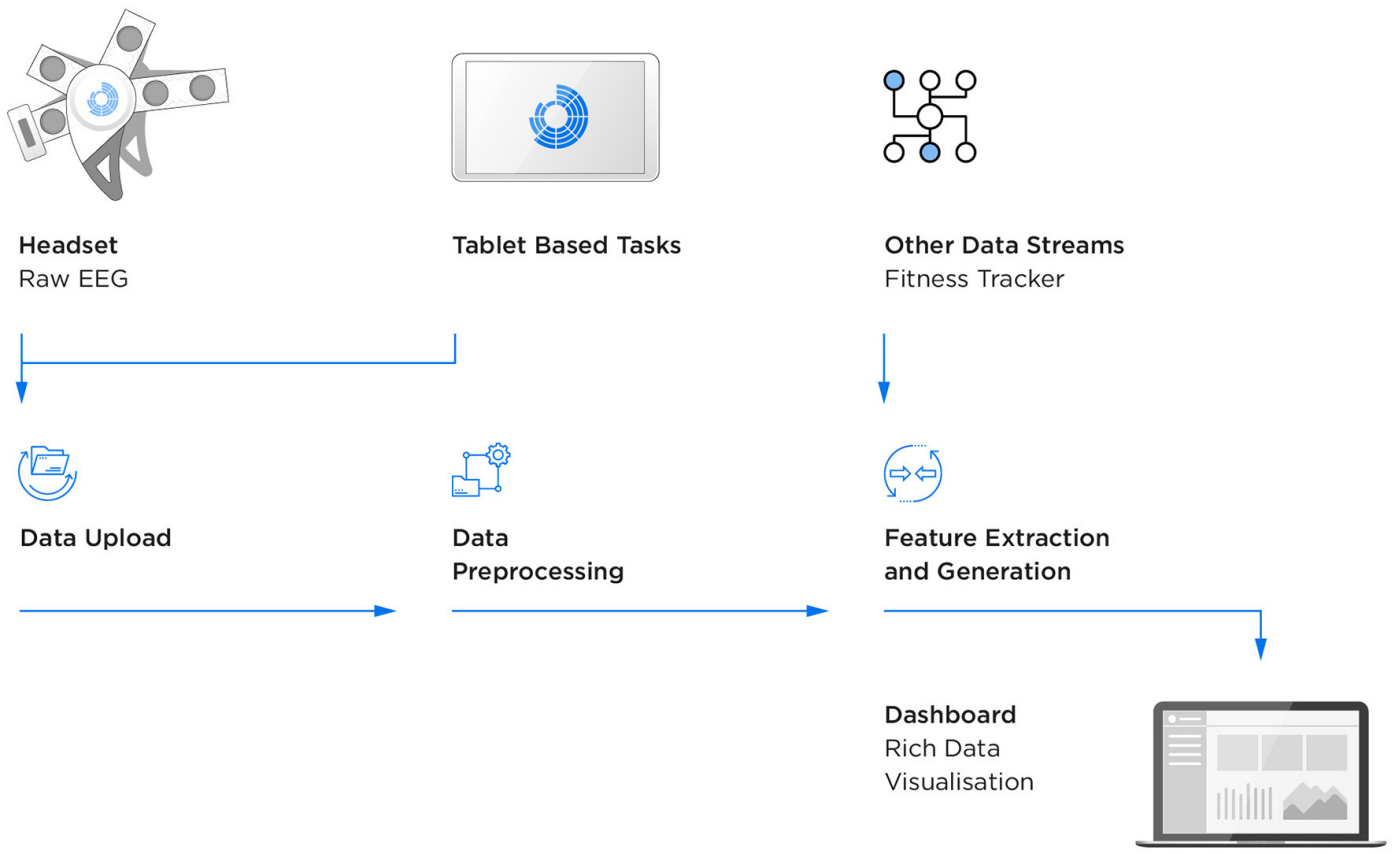
Flow of EEG and behavioural data.

The platform was designed to enable frequent, objective sampling of brain-based markers of cognition inside and outside of the clinic/lab setting, using a dry sensor, wireless encephalography (EEG) headset that records brain activity, accompanied by gamified versions of cognitive tasks presented via a tablet-based app. Upon logging into the app, a stepwise tutorial guides the user through setup of the headset (covering placement on the head, positioning of the detachable mastoid sensors and feedback on sensor impedances) in preparation for recording data during the gamified tasks. Cloud-based secure methods are used for collection and automatic processing, as well as integration with other data streams (in this study participants wore a fitness tracker, the Withings Go) and web-based dashboards for monitoring and data visualisation on a daily, session-by-session basis (Figure 1).

#### EEG

**Figure 2:**
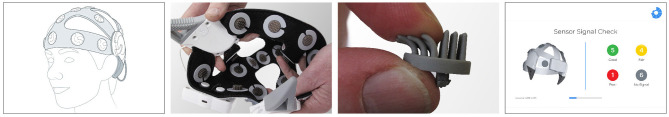
Sixteen-channel wireless headset designed with pliable sensors and the sensor signal quality check.

The wireless EEG headset (Figure 2) consists of dry flexible Ag/AgCl coated polymer sensors at 16 channels (O1, O2, P3, Pz, P4, Cz, FT7,FC3, FCz, FC4, FT8, Fz, AF7, AF8, FPz). The left and right mastoids are used for reference and driven-bias, with single-use, snap-on electrodes attached to wires extending from the headset. The electronics and sensors are mounted on flexible neoprene, and the stretchable structure incorporates anatomical landmarks in the form-fit of the headset to encourage consistent placement by users in line with the 10–20 sensor system. The analogue headset has high input impedance of 1 GΩ, a configurable driven bias function for common-mode rejection, built-in impedance checking, and configurable gain and sampling rates. An onboard processor and Bluetooth module transmit 250 Hz EEG data to the tablet, from where it is transferred to a cloud server for storage and processing. EEG recording and behavioural events are timestamp synchronised to ±2 ms.

#### Cognitive Tasks

**Figure 3:**
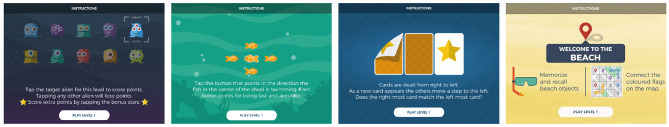
Images of 2-stimulus visual oddball, flanker, n-back, and delayed match-to-sample gamified tasks.

The gamified tasks (Figure 3) are based on well-known paradigms from experimental electrophysiology and cover a range of core cognitive functions. Cognitive/electrophysiological tasks were gamified with the aim of improving motivation, i.e., to enhance attentiveness during testing and to prevent boredom over repeated plays, while maintaining the core cognitive components of the original task. Feedback on gameplay performance was provided (e.g., points awarded for speed of responses where appropriate), along with personalised leaderboards to promote long-term adherence to the study schedule. Each daily session comprised a resting state plus two of the four other games (alternating between sessions). Participants also answered a daily health and lifestyle questionnaire to further contextualise the daily recordings. Daily sessions were designed to take <30 min total time from start to finish.

##### Two-Stimulus Visual Oddball

This gamified version of the classic 2-stimulus visual oddball paradigm (58), presents target stimuli (aliens—requiring the participant to tap on the screen, *n* = 30) and non-target stimuli (visually different aliens—requiring no response from the participant, *n* = 70) across five levels of gameplay, as well as 15 “bonus” stars throughout the game (to enhance gameplay and not included for analysis). Behaviour (reaction time and response accuracy) and corresponding EEG correlates are indicative of neural dynamics of the decision-making process and the attention and working memory on which it relies (59–61). Using EEG, a positive voltage deflection can be observed over the parietal cortex starting ~300 ms following presentation of the stimulus, known as the P300 event-related potential (ERP). With advancing age, the amplitude of the P300 is known to decrease, and its latency known to increase (62–64).

##### Flanker

Inhibition and error awareness were probed using a gamified version of the Erikson Flanker task (65). Fish served as directional stimuli and were presented across five levels with a shoal of fish (flanking stimuli) appearing first, followed by the central (target) fish. The participant was asked to tap on the side of the screen corresponding to the direction of the central fish, ignoring the flanking fish (either congruent or incongruent stimuli, split evenly between the 150 trials), presenting a cognitive challenge reflected in behavioural responses (accuracy and reaction time) and EEG. The relevant EEG metric extracted from this task is the Error-related negativity (ERN)—a negative voltage deflection observed on error trials most prominently over the fronto-central scalp, followed by a subsequent positive rebound in the signal (the Error Positivity, or Pe) (66). Previous studies have consistently reported a decrease in the negative amplitude of the ERN with progressing age (67).

##### N-Back

The visual n-back paradigm (68) taxes working memory and executive function with age-related differences in behavioural performance, according to recent meta-analysis (69). In the current study, this game had a continuous short-sequence memorisation of 4 different playing cards where the participant was asked whether the current card is a “match” or “no-match” to the card seen 2 trials before. This 2-back paradigm consisted of 100 trials presented across two levels, with a 33% match rate.

##### Delayed Match-to-Sample

A visual delayed match-to-sample task, this task targets recognition memory, a key cognitive function known to be affected by age (70), across 50 trials, presented in blocks of 5, with 50% overall match rate (71). Each level is set in a specific location (beach, jungle, etc) where the user is presented with a variety of objects which must be encoded into memory to be retrieved after a brief (10-s) distractor game which involves connecting dots. Points are earned by identifying previously presented items at retrieval and rejecting unseen items.

##### Resting State

In this passive task (72) participants selected a relaxing scene (forest, park or beach) for 1 min of restful eyes open followed by 1 min eyes closed. This task elicits resting electrocortical activity and seeks to produce an increase in the neural oscillatory power of the alpha frequency band (7–13 Hz) when eyes are closed relative to eyes open, a physiological measure sensitive to a range of neurocognitive and psychiatric disorders, ageing, as well as sleep quality and caffeine intake (22, 73–75).

### Procedure

Participants attended two in-lab sessions, at baseline and following 12 weeks of at-home use of the platform.

#### Lab Sessions

Lab sessions consisted of neuropsychological testing followed by in-lab use of the platform. Neuropsychological assessment was carried out for screening and to facilitate potential longitudinal follow-up and/or comparison with other datasets, and is not analysed in this paper. The MoCA (76) was selected as a screening tool using a cut-off of ≥24 to be representative of normal cognitive function, in line with findings reported in the Irish older adult general population (77, 78). Participants then completed tasks from the Cambridge Neuropsychological Test Automated Battery (CANTAB) covering multiple domains including psychomotor skills, executive function, memory and attention domains before completing a session with the platform. Participants provided ratings of the usability of the platform at baseline, 6 and 12 weeks into the study on the System Usability Scale (SUS), a 10-item industry standard questionnaire designed specifically to assess use of technology (79).

#### Home Sessions

Participants were asked to use the platform as described in section The Platform, at home, over the course of 12 weeks. These sessions were ~25–30 min and participants were asked to contribute 5 sessions per week (one session per day, days unspecified for participant convenience). Throughout the 12-week period, participants wore a fitness tracker to monitor their step count and sleep, and answered questions about daily well-being and lifestyle habits (not analysed in the current paper).

### Analysis

To measure usability of the platform across age groups and feasibility of extracting features reflecting cognitive ageing, participants were assigned to three groups for analysis: those aged 40–54 (*n* = 26), 55–66 (*n* = 35), and 67–79 (*n* = 17) years. Usability measures used for analysis were adherence and participant-reported SUS scores, as well as technical measures of signal reliability. To investigate feasibility and explore effects of cognitive ageing, behavioural and EEG metrics were extracted across age groups. Additionally, event-related potential waveforms were plotted for comparison based on single game-play median epoch, single-participant averaged epochs and whole-sample grand averaged epochs. Validity of the approach can be established by confirming that behavioural and neural patterns observable in the literature (e.g., differences in timing between congruent and incongruent trials; the waveform and scalp topography of ERP components) are seen in the data recorded unsupervised in the home, and that age-related changes in these variables reflect the published consensus. Ninety five percentage confidence intervals are reported throughout using the upper and lower bounds.

#### Behavioural Analysis

Measures of accuracy and speed of response were extracted from the cognitive games played using the platform (2-stimulus oddball, flanker, n-back and delayed match-to-sample) to establish face validity against that which the literature leads us to expect. For this analysis, we averaged different behavioural measures across game-plays. To investigate reaction times, we chose the median reaction time per game-play, taking the median-average per participant to calculate age group mean comparisons and sample means. To compare rates of accuracy, we calculated percentage accuracy per game-play. We produced a mean accuracy rate per participant for age group and sample mean comparisons. Additionally, we calculated confidence intervals as an indication of variance. To visualise age group differences across game plays, the log-transformed game-play number was used as an explanatory variable of the different behavioural metrics per group in a linear model and a 95% confidence interval was calculated using 1,000 bootstrapping resamples.

#### EEG Analysis

The processing pipeline consists of filtering from 0.25 to 40 Hz, customised artefact removal, epoch extraction and baseline removal. Metric-based methods for removing invalid ERPs and PSDs were applied to outputs. Two event-related potential (ERP) components, the P300 (a positive-going waveform which peaks >300 ms after the presentation of an attended stimulus, associated with decision-making) and the Error-Related Negativity (a negative-going, response-locked waveform associated with error-awareness) were computed as the smoothed pointwise median of epochs within each session. Power spectral density (PSD) was computed using a 1,024 point Fast Fourier Transform (FFT) with Welch's method of averaging (using a 256 sample window) on the resting-state eyes-open and eyes-closed data. For this analysis, time-series data was converted to average reference to remove lateralised effects of the original single-mastoid reference. Mean and 95% confidence intervals were computed across all sessions from participants within each age group.

## Results

### Usability

Of the 89 healthy adult participants that enrolled in the study (67 female, mean age = 58.78, mean MoCA score = 27.12), 11 participants withdrew and 78 (61 female) completed the study (mean age = 58.99 mean MoCA score = 27.06), yielding an attrition rate of 12.40%. Data from those who withdrew was excluded from the following analyses reported.

Reasons for withdrawal cited were work/caring/other commitments (*n* = 3) and/or illness/health-related issues (*n* = 6). Two participants cited both health and caring commitments. One participant mentioned a faulty headset as an additional factor in the decision to withdraw; this participant's headset had required repair. Four participants did not give any reason. The mean number of sessions contributed by participants who withdrew was 16.82 [8.41–25.23], ranging from 1 (completed in-lab) to 45 sessions. The mean duration of at-home involvement by those who withdrew was 5.55 weeks [3.22–7.88].

**Figure 4:**
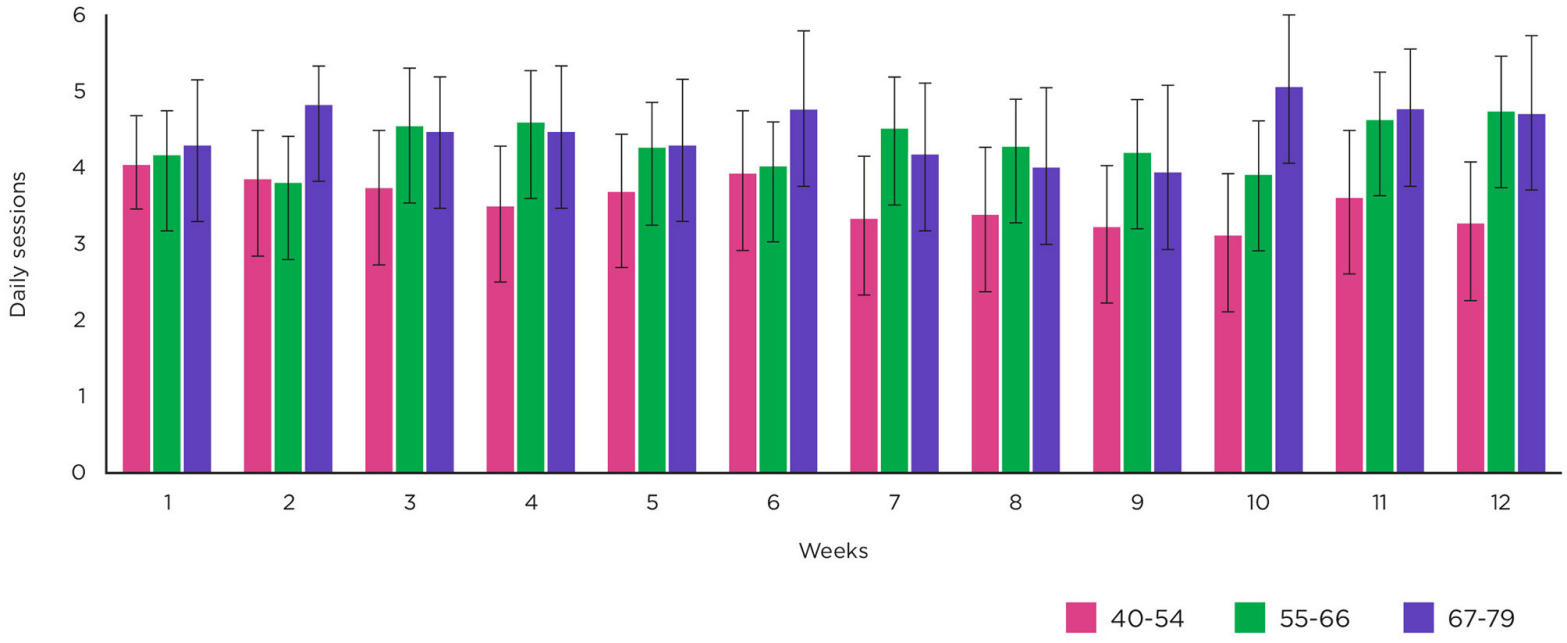
Weekly adherence. Mean number of sessions per week across all participants and by age group. Error bars show upper and lower 95% confidence intervals.

Figure 4 shows the rate of weekly adherence for those who completed the study (*n* = 78), including a breakdown of weekly adherence by age group [40–54 years (*n* = 26), 55–66 years (*n* = 35) and 67–79 years (*n* = 17)]. For those who completed, mean number of sessions contributed per week was 4.10 [3.97–4.23], out of a target of 5 per week and the mean total number of sessions contributed per participant was 49.14 [46.54–51.74]. By age group, mean number of sessions per week was 3.56 [3.33–3.78] for those aged 40–54, 4.31 [4.12–4.50] for those aged 55–66 and 4.48 [4.22–4.74] for those aged 67–79 years.

Participants were asked to evaluate usability via the System Usability Scale (SUS) at 3 timepoints. Mean SUS scores were 76.59 [72.94–80.24] at baseline, 81.45 [78.33–84.57] at 6 weeks and 78.28 [74.74–81.81] at 12 weeks (see Table 1). It is worth noting that the mean SUS score at baseline from those who subsequently withdrew was 75.23 [64.42–86.04] and of those who were still enrolled at 6 weeks, mean SUS score was 74.64 [62.62–86.67].

**Table 1:** System usability scale scores by age group at baseline, 6 and 12 weeks.

	**40–54 years**			**55–66 years**			**67–79 years**		
	**Mean**	**95% CI**	***n***	**Mean**	**95% CI**	***n***	**Mean**	**95% CI**	***n***
Baseline	81.35	±5.99	26	77.94	±4.60	34	66.62	±9.01	17
6 weeks	81.56	±5.81	24	85.44	±3.69	34	72.81	±7.08	16
12 weeks	80.50	±6.04	25	81.50	±4.62	35	68.38	±8.05	17

**Figure 5:**
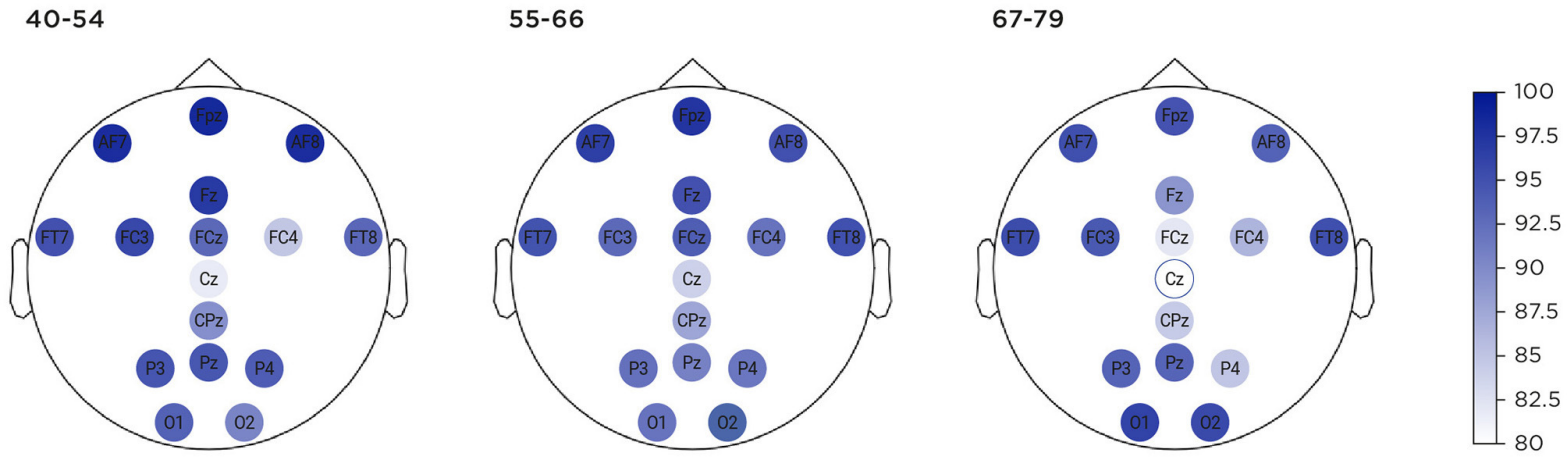
Percentage of time each of 16 channels recorded non-saturated data, shown across age groups.

Ability to use the system to record usable EEG in the at-home setting, reported in Figure 5, was considered by measuring the percentage of time that individual sensors were connected to the scalp (i.e., recording non-saturated data) for the different age groups. Three thousand six hundred three sessions were successfully uploaded to the cloud server. Of these, 95.81% (3,452 sessions) contained portions of EEG data that could be used for analysis, even though certain sections of that session, or certain sensors, may be very noisy. One hundred and fifty-one sessions were rejected in their entirety, due to saturated data sections, high variance sections or gaps. By comparison, the behavioural data, where 99.03% (3,568 sessions) contained a complete set of response measures for at least one of the two games assigned per session. There were 116 sessions for which behavioural data, but not EEG data, was suitable for analysis.

### Behaviour

**Figure 6:**
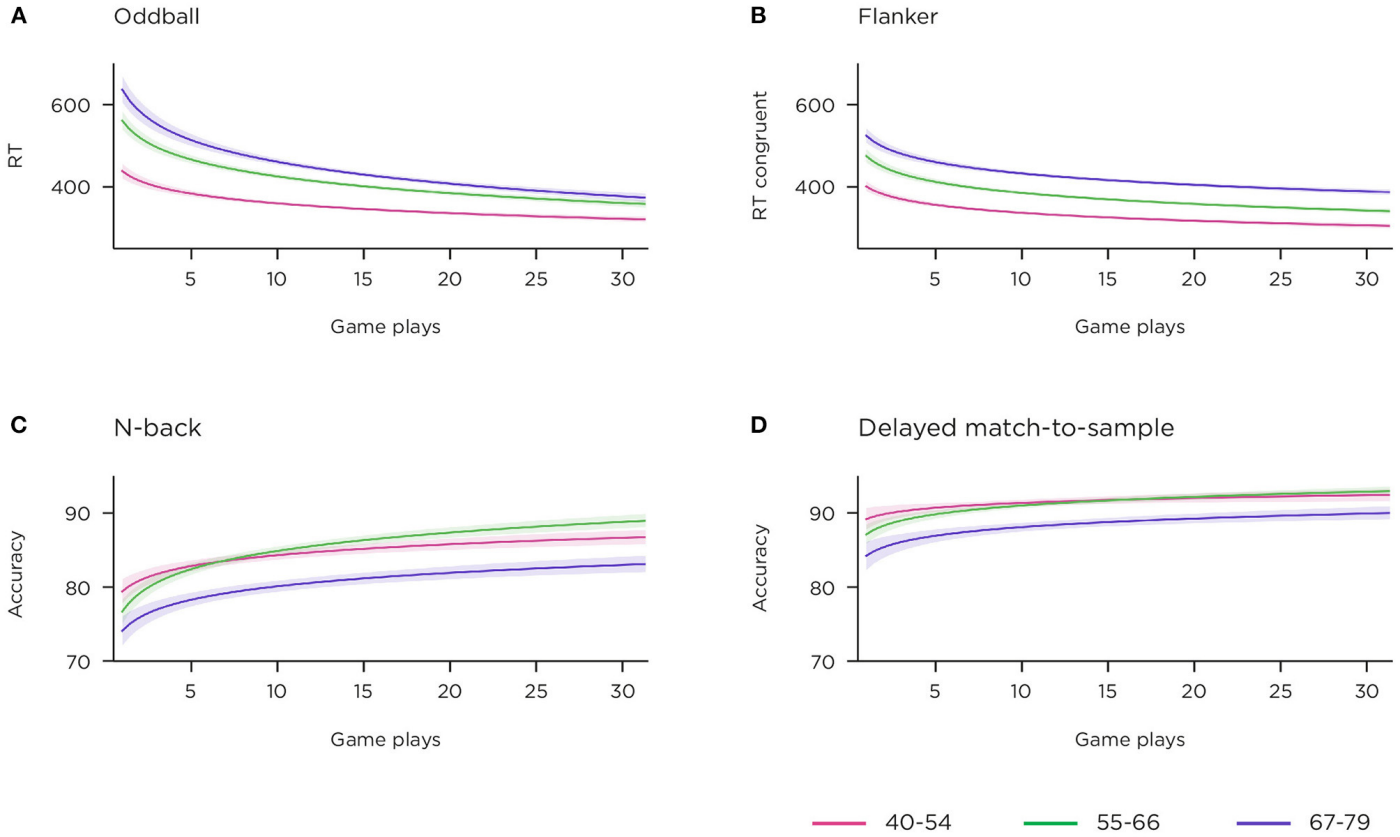
Behavioural responses to gamified cognitive tasks over 12 weeks across age group. Shading indicates 95% confidence interval. **(A)** median correct RTs to targets in 2-stimulus oddball task; **(B)** median correct RTs to congruent trials in flanker task; **(C)** percentage accuracy, all trials, n-back task; **(D)** percentage accuracy, all trials, delayed match-to-sample task.

To establish face validity of the gamified behavioural tasks, key metrics from each game were extracted to evaluate against what would be expected from traditional lab paradigms described in the literature. The temporal development of several illustrative metrics, stratified by age-group, is displayed in Figure 6.

#### Two-Stimulus Oddball Game

Similar to lab versions of simple decision-making task which do not challenge the accuracy of responses, the gamified version demonstrated high accuracy, mean 97.71 [97.08–98.35]%. Age group means were 98.7 [98.32–99.08]% for 40–54 years, 98.05 [97.16–98.95]% for 55–66 years, and 95.5 [93.62–97.39]% for 67–79 years. As the game rewards speed of response (in its scoring procedure), and due to learning/strategizing effects, we expected reaction time (RT) to improve with repeated gameplays. Figure 6A shows the temporal trend of speed of response over consecutive sessions, and clear separation of the three age bands is visible. Reaction time per group, averaged over all sessions, was 366.76 [352.68–380.84] milliseconds (ms) for 40–54 years, 414.85 [393.17–436.54] ms for 55–66 years and 449.58 [423.45–475.70] ms for 67–79 years.

#### Flanker Game

This task is time-restricted, and encourages the player to make a trade-off between speed and accuracy of response (as erroneous response trials are those that yield the key EEG metric). Incongruent trials require inhibition and possible motor-replanning relative to congruent trials, and this is seen in an inhibition cost of 62.16 [56.62–67.7] ms. The inhibition cost per age group was 55.87 [48.28–63.45] ms for 40–54 years, 64.67 [56.0–73.34] ms for 55–66 years and 66.89 [52.99–80.8] ms for 67–79 years.

Incongruent trials induced many more errors [8.45 (7.06–9.84)] than congruent trials [2.02 (1.66–2.68)]. Looking at the temporal development of the congruent condition reaction times alone (Figure 6B), a pattern of learning from session to session, and separation of age bands, is visible.

#### N-Back Game

This game requires cycling of information in and out of short-term memory. RTs were slower for non-match [1,075.83 (990.77–1,160.9) ms] than match trials [919.56 (857.76–981.36) ms], however accuracy rates were higher for non-match [87.71 (86.41–89.01)%] vs. match trials [74.76 (71.91–77.60)%]. Accuracy rate was 76.49 [72.96–80.02]% for 40–54 years, 75.56 [70.57–80.55]% for 55–66 years and 70.46 [64.6–76.32]% for 67–79 years on match trials and 87.51 [85.73–89.29]% for 40–54 years, 89.29 [87.41–91.16]% for 55–66 years and 84.77 [81.47–88.08]% for 67–79 years on non-match trials. Figure 6C shows accuracy rates on all trials, by age group, across repeated game-plays.

#### Delayed Match-to-Sample Game

This is not a speed challenge task, however, RTs to match trials were faster than non-match trials 886.99 [847.27–926.71] ms vs. 1,060.31 [1,013.4–1,107.22] ms, a pattern reflected in RTs by age group: 796.27 [754.02–838.52] ms, 890.05 [840.75–939.35] ms and 1,027.89 [914.95–1,140.83] ms for match trials compared to 944.11 [898.82–989.41] ms, 1,079.58 [1,007.6–1,151.56] ms and 1,208.17 [1,102.96–1,313.39] ms for non-match trials, for 40–54, 55–66, and 67–79 years. Memory performance is known to decrease with age, and divergence can be seen in Figure 6D for the oldest age-band, though again not between the younger and middle bands. Separate examination of the accuracy for match and non-match trials showed that this difference in performance was primarily driven by the non-match trials. Over the sample, accuracy was 93.16 [92.04–94.28] for match vs. 81.82 [79.35–84.30] for non-match trials, while accuracy rates across the age groups showed more difference for non-match compared to match trials: 85.67 [82.61–88.72], 80.98 [76.72–85.24], and 77.38 [72.51–82.24], compared to 93.85 [92.41–95.28], 93.12[91.24–94.99], and 92.12 [89.44–94.81] for non-match and match trials, respectively, for the age groups 40–54, 55–66, and 67–79 years. These results suggest that the non-match trials acted as effective lures.

### EEG

**Figure 7:**
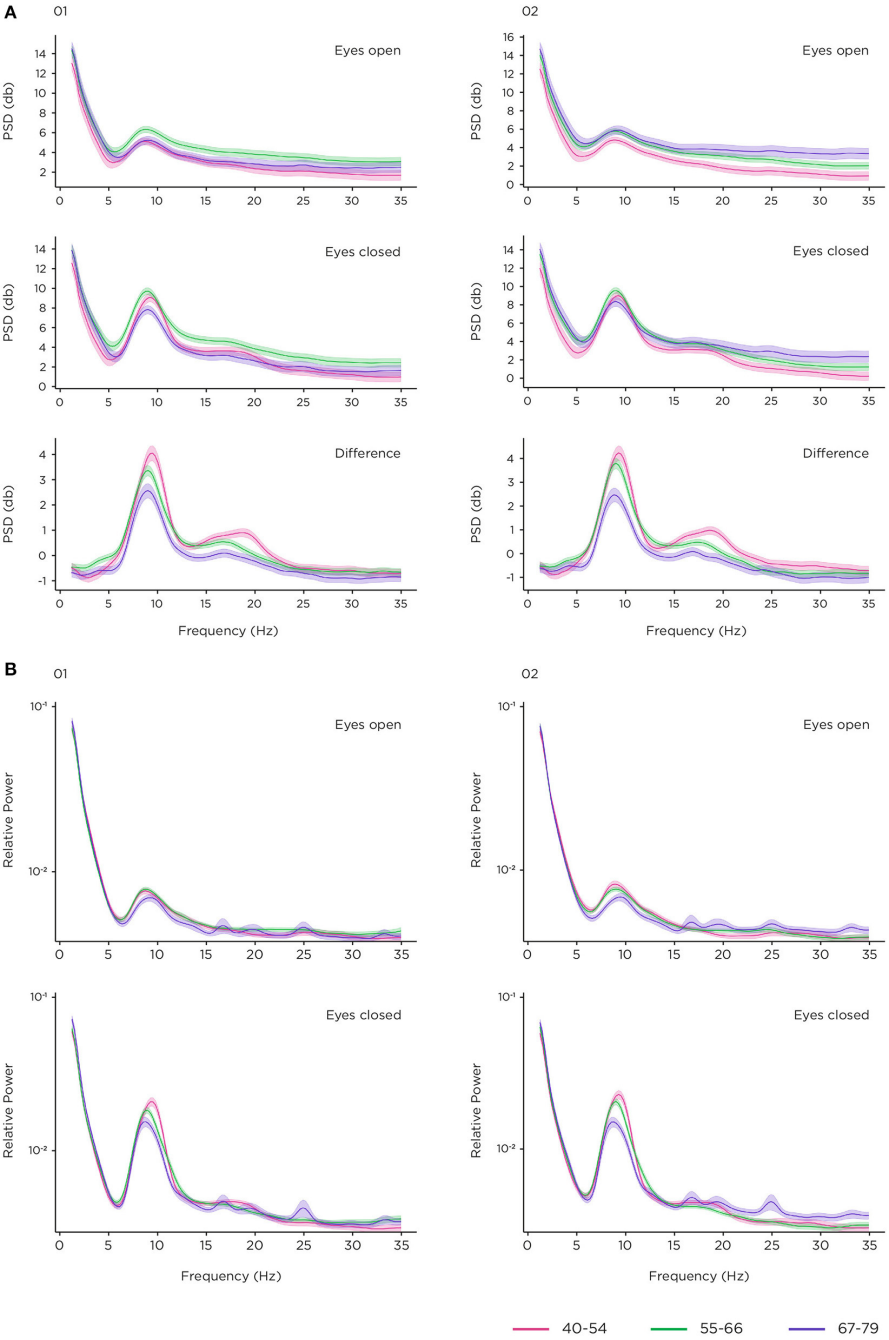
Resting state task. **(A)** power spectral density (PSD) in decibels (dB) at O1 and O2 by age group in eyes-open and eyes-closed conditions, and the difference condition; **(B)** relative power at O1 and O2 by age group in eyes-open and eyes-closed conditions with logarithmic scaling for display only.

For the resting-state task, power spectral density (PSD) was plotted at occipital sites to explore the effectiveness of the platform to measure change in alpha power between the eyes-open and eyes-closed conditions of resting-state task, across the three different age bands (Figure 7). Data from all 78 participants was included in the analysis. The number of sessions per comparison at electrode site O1 for eyes-open/eyes-closed were 904 for 40–54 years, 1,511 for 55–66 years, and 791 for 67–79 years. For electrode site O2, number of sessions were 903 (40–54 years), 1,513 (55–66 years) and 792 (67–79 years). The eyes-open data clearly shows the expected 1/f pattern of signal power falling with increasing frequency, and an alpha band peak around 10 Hz. As expected, the alpha peak amplitude is increased in the eyes-closed condition, as well as in the lower beta band (15–20 Hz). Figure 7A displays the effect of age group on absolute band power. There is a clear monotonic decrease in power with age in the difference condition with the largest eyes-open/eyes-closed difference for those aged 40–54 and the smallest difference for those aged 67–79 in the alpha and lower beta range. Furthermore, it can be seen that the average peak alpha frequency is highest for younger participants, and lowest for older participants. No consistent pattern is apparent in alpha power for the eyes-open and eyes-closed conditions alone, although there are clear distinctions between groups in the gamma range (30–35 Hz). This may indicate a difference in noise floor between the age groups. We applied a suitable normalisation by taking the relative power on this analysis (80). Relative power is shown in Figure 7B, again demonstrating a stratified pattern of age group on alpha power and peak frequency, most evident in the graph of the eyes-closed condition. It is noticeable that there is more fluctuation in the higher frequencies for the oldest age group (67–79 year olds).

**Figure 8:**
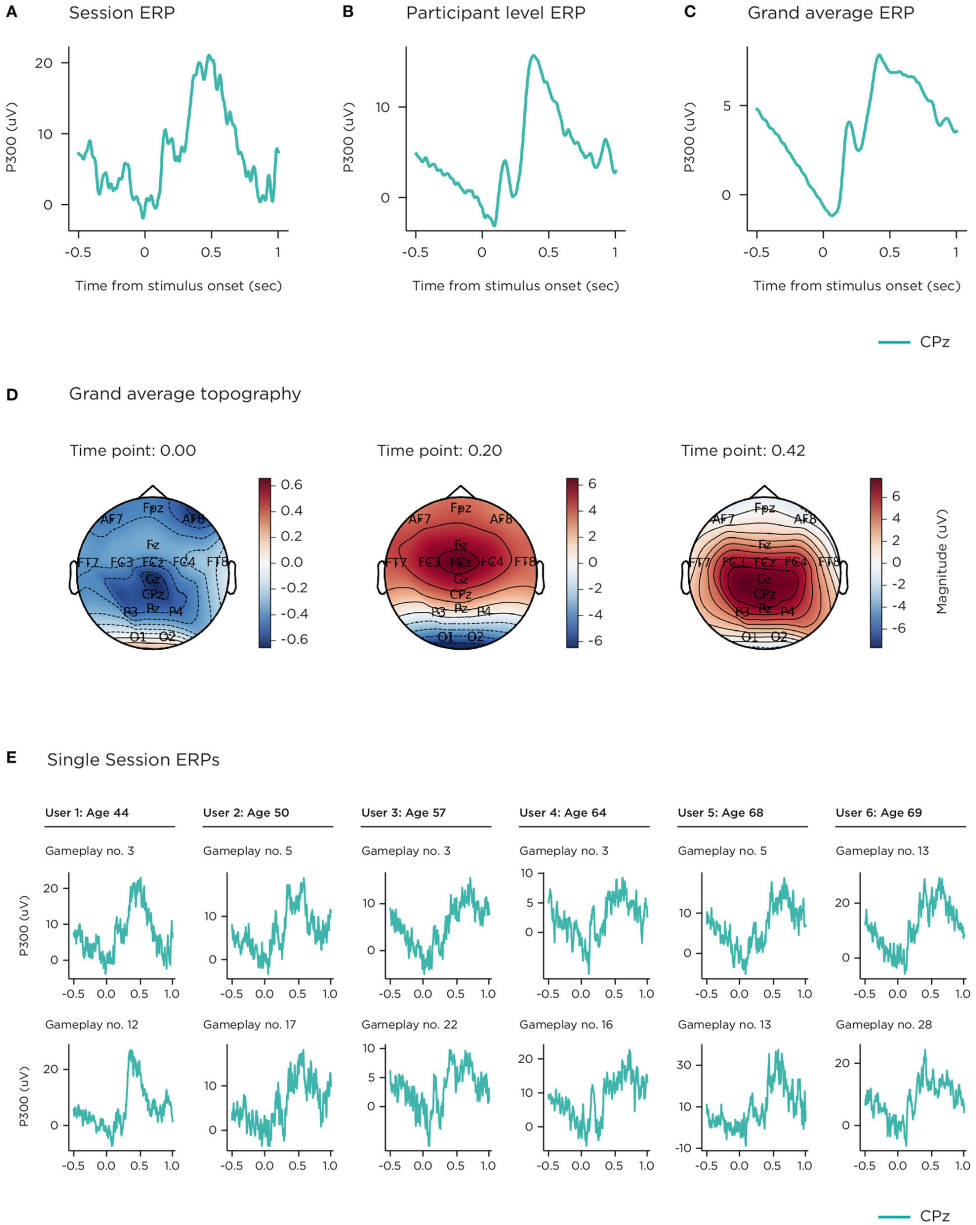
P300. **(A)** single-session median; **(B)** single-participant mean; **(C)** grand mean; **(D)** grand mean topographies selected at ERP peak timepoints; **(E)** examples of single-session median ERPs successfully recorded from game plays from 6 different participants (2 participants per age group).

**Figure 9:**
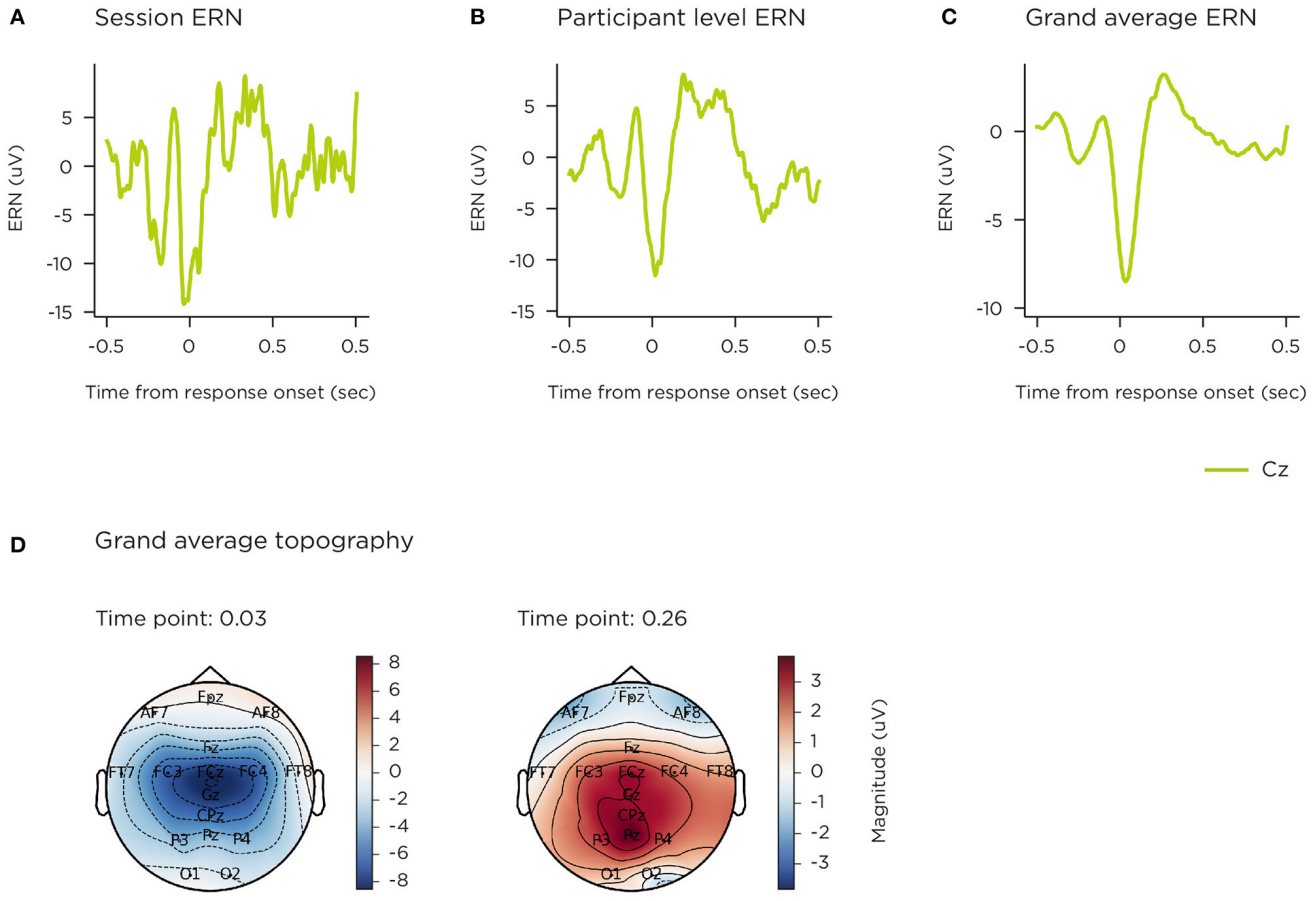
ERN. **(A)** single-session median; **(B)** single-participant mean; **(C)** grand mean; **(D)** grand mean topographies selected at ERP peak timepoints.

Evoked and event-related potentials elicited in the gamified 2-stimulus oddball and flanker tasks were also extracted at a single-session, single-participant and grand average level (shown in Figures 8, 9). Figure 8C illustrates the grand average ERP for target trials on the 2-stimulus oddball, at the centro-parietal location CPz, where the P300 is centred. This is a robust average over multiple sessions contributed by 77 participants, time-locked to the presentation of the stimulus (data from one participant, *n* = 26 sessions, did not meet quality thresholds for inclusion at this channel). Interpolated topographies (Figure 8D) across all 16 channels at ERP peaks are shown at 0, 200 and 420 ms post-stimulus onset. The principal waveform features of a P300 ERP are visible in the early sensory processing components (~0–250 ms with an occipital focus) and the P300 component (~300–500 ms, with a centro-parietal focus). A strong readiness potential can also be seen before stimulus presentation (−500–0 ms). The other two panels show the median stimulus-locked epoch from 29 correctly identified target trials from a single game-play session (Figure 8A), and the median-average across 18 out of a total of 21 game-play sessions (3 did not meet quality thresholds), contributed by a participant aged 44 years (Figure 8B). Figure 8E demonstrates examples of successful session-level ERPs evoked during a single game-play session, representing 6 users across the different age groups in the study (2 participants from each age group). Unsuccessful sessions yield waveforms that show a discernible ERP overlaid with noise, flat-line signals (e.g., due to an unconnected sensor), or noise of various heterogenous types.

Figure 9C illustrates the grand average difference ERP for error trials on the flanker task, at the central Cz location, where the ERN is observed (total 1,004 sessions). This is a robust average over multiple sessions contributed by 76 participants, time-locked to touch-response (single-channel data was excluded from 2 participants, who contributed 6 and 8 sessions). Grand average topography (Figure 9D) across all 16 channels at ERP peaks are shown at 30 and 260 ms post-stimulus onset. The event-related negativity (ERN) waveform is clearly represented (peaking at ~50 ms), with a central focus, as is the error positivity (Pe) with a centro-parietal focus (peak ~250 ms). Figure 9A shows the corresponding median response-locked epoch across 12 error trials following a single game-play session, contributed by a participant aged 52 years. Figure 9B displays that participant's median-average across 14 out of 16 game-play sessions.

**Figure 10:**
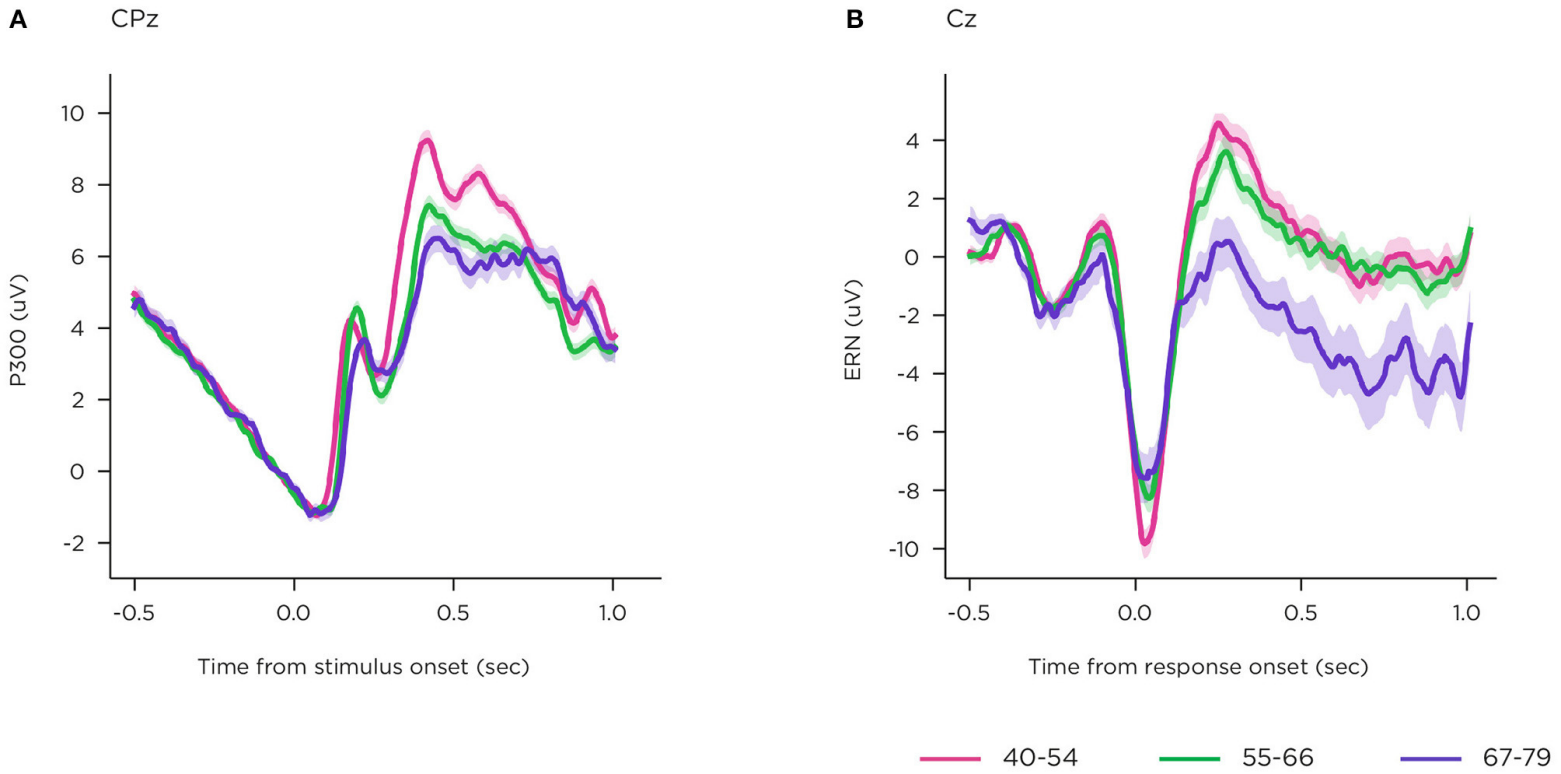
Showing age-related differences in event-related components recorded using the platform. **(A)** P300; **(B)** ERN.

The P300 and ERN components were also compared by age group. Figure 10A shows grand-averaged epochs per age group on single channel CPz for the stimulus-locked ERP from the visual oddball, and Figure 10B shows these at Cz for the response-locked ERP of the flanker task. The number of participants and sessions per comparison at electrode site CPz were 26 and 397 for 40–54 years; 34 and 515 for 55–66 years; 17 and 240 for 67–79 years. For electrode site Cz, number of participants and sessions were 23 and 339 (40–54 years); 35 and 473 (55–66 years); 17 and 182 (67–79 years). The impact of signal variability from individual sessions (both noise, and genuine inter-individual differences) is quantified in the 95% confidence intervals illustrated.

The P300 shows early differences in latency in sensory processing, and separation in amplitude among the groups, where disruption increases with age. In the Flanker task ERP, the early ERN component is attenuated in both older groups, relative to the youngest group, and the later Pe component is reduced for the oldest group.

## Discussion

### Usability

This proof-of-concept paper reports findings from the first time that this novel EEG platform was deployed in-field. After a single training session, participants, including older adults up to the age of 79 years, were able to use the technology at home to successfully perform EEG and behavioural recordings without the supervision of trained technicians. This study yielded 3,603 uploaded sessions, >95% of which contained usable data (i.e., EEG and behavioural metrics could be extracted from the submitted data), providing encouraging evidence supporting the feasibility of this technological approach to cognitive neuroscience research.

Users were asked to trial and evaluate this new system for monitoring brain health at home and to contribute five 30-min recording sessions per week, which was a considerable effort given that no extrinsic incentives or disincentives were applied to promote adherence to this schedule. Adherence to this schedule was remarkably high, relative to other reports of in-home monitoring devices in older populations over similar time courses [~55% (81, 82)]. For the current study, attrition was low (12.4%) and the average contribution was >4 sessions per week (>80% adherence to schedule). Older adults had the highest rates of adherence, indicating that age was no impediment to using the system regularly. A high level of adherence was maintained throughout the 3-month period without substantial decline in the latter weeks of participation, testament to both the power of gamification and usability of the system motivating and facilitating repeated play, and the level of commitment from the study participants. Maintaining adherence in unsupervised environments is challenging and may be particularly so for psychiatric populations (83), however widespread evidence from other diseases, where there has been a broad uptake in new technologies, indicates that patient-centric digital monitoring provides more objective, frequent tracking with clear healthcare benefits (84–86) and have been shown to lead to better compliance relative to paper based assessments (86, 87).

Reported usability was somewhat lower for the oldest age-band, although it is worth noting that their lowest average score still falls within the range between “ok” and “good” (88). Contrary to our expectations, this did not result in reduced adherence, suggesting that many challenges of manual dexterity or familiarity with digital technologies had been successfully mitigated during the initial user-focused design process. However, signal reliability measures indicated that the oldest age band experienced the greatest difficulty in obtaining good sensor connectivity, particularly around the midline sensors (Cz) located at the top of the head. Younger participants achieved slightly better connectivity at Cz but also better connectivity on adjacent sensors. Head shape is variable at the crown, meaning that generic headset sizing options are not always optimal. That location may also require additional manual dexterity and adjustment, which is more difficult for older populations. In the light of these findings from the first deployment of this technology in-field, subsequent incremental improvements to the headset, app and enrolment training procedures have been deployed which have resulted in superior sensor connectivity and data quality (89, 90).

In addition to investigating the overall usability of the platform, empirical data collected in this study was used to assess the potential of collecting scientifically valid neurocognitive data from remote, fully autonomous participants.

### Behaviour

All gamified cognitive tasks exhibited some degree of a learning effect. Reaction times generally decreased rapidly over the first five sessions of a given task (see Figure 6). This likely reflects the development of task-specific perceptual-motor skill, rather than a change in the underlying cognitive function probed by the task (i.e., “brain training”). Time spent developing task strategies, and learning the layout of the task environment, is likely to have enabled more effective allocation of visual attention and therefore faster responding (91). Age-related effects on speed of response were generally preserved throughout this learning phase and into asymptote, with the oldest participants consistently making their responses more slowly than other age groups, consistent with the literature. Regarding accuracy over time, participants on average improved very slowly and consistently on the n-back task throughout the study, as expected since n-back variants are typically included with brain training suites due to their inherent learnability (92). Participants, regardless of age band, demonstrated less of a learning effect in the visual delayed match-to-sample task, potentially reflecting the simplicity of this basic old/new matching task for healthy adults however again, the oldest participants scored lowest on this task throughout the study, consistent with age-related decline in memory performance. These learning curves themselves (Figure 6), enabled through the ability to collect multiple assessments over time, may be a rich source of data and potentially informative measures of underlying cognitive function (93), with recent research showing that rate of learning, in the context of a cognitive task conducted over multiple days, can differentiate groups by age (94) and neuropathology (95).

### EEG

EEG signals described in this paper (resting-state spectral activity and the P300 and ERN ERPs) demonstrate morphology consistent with those elicited by non-gamified, laboratory paradigms described in the literature. Furthermore, grand average visualisations of P300 and ERN ERPs across age bands replicate classic electrophysiological patterns of age-related change.

The study design included repeated use to permit aggregation of EEG data collected in the home, as a means of improving reliability and signal to noise ratio. The focus in this paper is on group level grand-average analyses, common in cognitive neuroscience literature. Although, as can be seen in the data presented in this paper, it is feasible to obtain cognitive ERP components from users (across different age groups) based on single, home-based sessions. As might be expected, not all sessions were available for analysis with factors such as saturated signal or high variance rendering the data unusable. However, over 95% of sessions contained EEG from which at least a portion of the data was usable, even though certain sections of that session, or certain sensors, may have been noisy. In order to support participants to achieve good signal quality, the system included a sensor signal check step at the beginning of every session to give feedback on impedance levels to encourage self-adjustment for a good connection.

EEG devices that offer miniaturisation of the EEG amplifier, use of Bluetooth technology to transmit EEG data, precise stimulus event-marking, and a choice of wet or dry sensor set-ups, have been extensively evaluated in the literature [e.g., (54, 96–99)]. These demonstrate reduced set-up times and greater portability while generally maintaining good signal-to-noise ratio [e.g., (52, 53, 100, 101); but see Duvinage et al. (102), Maskeliunas et al. (103)]. However, the authors are not aware of reports of any other mobile EEG system for which repeated ERP data collection has been demonstrated in participants' home environment without a researcher present, as was the case in this study.

The P300 elicited from the 2-stimulus oddball task exhibited reduced amplitude and latency for the older age groups, consistent with previous studies (62–64). Whilst the underlying mechanisms are yet not fully elucidated, recent evidence points toward the P300 reflecting the accumulation of information leading to a decision (60), the ability to do this effectively being impaired by ageing and cognitive decline (104). ERP components evoked from the Flanker task also demonstrated sensitivity to ageing with a smaller ERN for the older age groups and a weak Pe for the oldest age band, reproducing known effects in the literature, reflecting a general weakening of the processes underpinning cognitive control in ageing populations (67, 105). Resting state EEG PSD demonstrated alpha band increase in the eyes closed condition relative to eyes open as expected. In the absolute power analyses differences in noise levels were observed. Grummet et al. (106) discusses variability in noise floor in dry EEG, which in this case may potentially be driven by factors such as systematic variation in the use of the headset, and age-related changes in skin condition (107). However, having controlled for differences in noise floor levels, we observed alpha band power and peak frequency stratification across that age groups that align with the effects of ageing on brain activity during resting state typically reported in the literature (74, 108, 109).

## Conclusion

In this paper we have described the first large scale field trial of a new suite of tools to collect clinically relevant domain-specific markers of brain function and cognitive performance unsupervised in the home. Human-factors feasibility was demonstrated by high reported usability, low levels of withdrawal, and adherence of >80% over a 5-day-per-week, 3-month long, uncompensated participation. Newly gamified versions of established tasks were trialled and were successful in replicating key aspects of behaviour from their lab-based counterparts. Widespread learning effects were observed, as would be expected on repeated plays, but age-related differences were preserved over many weeks of repeated play. Grand average EEG data from the resting state, visual oddball and flanker tasks all illustrated core features of frequency content, waveform morphology and timing, and scalp topographies to confirm that they faithfully replicate the lab-based tasks on which they were modelled.

Challenges of data quality were encountered. On an average session, 14 or 15 sensors (of 16) provided EEG signals that could be analysed, the remainder lost due to issues with contact reliability with particular scalp locations and age cohorts. Certain sessions were evaluated as too noisy for inclusion in grand average analyses and early behavioural sessions proved more variable than later ones. Since this study was completed, incremental improvements to the headset, tablet-based app and participant familiarisation procedures have been made that have increased signal quality (89, 90).

While the focus of this paper is on ageing, and cognitive functions of relevance to Alzheimer's disease and other pathologies underlying dementia, this suite of tools can also include additional tasks (e.g., emotional face processing, passive auditory oddball) suitable for use in mood disorders, psychosis (110), and measurement of treatment response in psychiatry (89, 111).

Advances in wearable electronics, dry sensors and user-facing interactive technologies enable EEG as an easy-to-use affordable biomarker of cognition, grounded directly in brain function. Decades of scientific literature support EEG as an emerging translational biomarker for disease cases in neuropsychiatric (schizophrenia, depression) (20, 112, 113) and neurodegenerative (e.g., Alzheimer's) disease (114–116). Sampling a broad suite of cognitive functions (including memory, attention, and executive function) offers coverage of multiple cognitive domains, which has greater predictive accuracy for disease progression (117, 118). Cloud computing can securely collect data from distributed locations, automatically evaluate quality, and use machine learning techniques to derive composite markers based on neural activity and behavioural performance from single and multiple cognitive domains (119). These innovations in technology, supported by scientific literature, make it possible to use large-scale longitudinal sampling of real-world data, to support potential future use-cases in early detection, personalised medicine, progression tracking, and measurement of treatment response for neuropsychiatric disorders.

## Data Availability Statement

The datasets presented in this article are not readily available because the data collected using the Cumulus Neuroscience platform is commercially sensitive and contains proprietary information. Requests to access supporting data will be considered from bona fide researchers upon reasonable request. Requests to access the datasets should be directed to alison@cumulusneuro.com.

## Ethics Statement

The studies involving human participants were reviewed and approved by Queen's University Belfast Faculty Research Ethics Committee (EPS). The patients/participants provided their written informed consent to participate in this study.

## Author Contributions

EM, BMu, BMc, PP, and AB contributed to the conception and design of the study and paper. AB performed data collection. EM, LR-D, HN, MI, JD, FB, BMu, and AB performed analysis and interpretation. EM, JD, BMu, and AB wrote the manuscript. LR-D, HN, and MI wrote sections of the manuscript. All authors critically revised the manuscript and approve the submitted version.

## Conflict of Interest

LR-D, AB, EM, JD, HN, MI, FB, and BMu are employees of the company Cumulus Neuroscience Ltd. The remaining authors declare that the research was conducted in the absence of any commercial or financial relationships that could be construed as a potential conflict of interest.
